# PAR-3 Knockdown Enhances Adhesion Rate of PANC-1 Cells via Increased Expression of Integrinαv and E-Cadherin

**DOI:** 10.1371/journal.pone.0093879

**Published:** 2014-04-03

**Authors:** Liora Segal, Liora S. Katz, Hagit Shapira, Judith Sandbank, Elizabeth Geras-Raaka, Marvin C. Gershengorn, Yoram Oron

**Affiliations:** 1 Department of Physiology and Pharmacology, Sackler Faculty of Medicine, Tel Aviv University, Ramat Aviv, Israel; 2 Laboratory of Endocrinology and Receptor Biology, National Institute of Diabetes and Digestive and Kidney Diseases, National Institutes of Health, Bethesda, Maryland, United States of America; 3 Institute of Pathology, Central Lab, Maccabi, Rehovot, Israel; 4 Assaf Harofe Hospital Institute of Pathology and the Department of Pathology, Sackler Faculty of Medicine, Tel Aviv University, Ramat Aviv, Israel; Thomas Jefferson University, United States of America

## Abstract

The balance between the adhesion of cancer cells to extracellular matrix and their migratory potential, as well as their proteolytic activity, are important parameters that determine cancer cells invasiveness and metastasis. Since thrombin has been implicated in cancer progression, we studied the role(s) of thrombin-activated receptors in the adhesion process. We stably knocked down proteinase-activated receptors (PARs) -1, or -3 in human pancreatic adenocarcinoma PANC-1 cells. PANC-1 cells exhibit rapid adhesion to cell culture treated plastic and much faster kinetics of adhesion to Matrigel coated surface. Knockdown of PAR-1 had no effect on cells' adhesiveness, while PAR-3 knockdowns (KDs) exhibited much faster adhesion kinetics. PAR-3 KDs also exhibited slower in vitro wound closure than vector-control and PAR-1 KD cells. To study the molecular mechanism(s) of PAR-3 KD cells' enhanced rate of adhesion, we assayed the expression of the molecules that mediate cell-surface and cell-cell adhesion. ITGαv, as well as ITGα6 and ITGα10 mRNAs, were greatly enriched (>40-fold) in a rapidly-adhering sub-population of PAR-3 KD cells. The whole population of both PAR-1 and -3 KDs exhibited enhanced expression of a number of integrins (ITGs) mRNAs. However, ITGαv mRNA and protein expression was increased in PAR-3 KD and markedly decreased in PAR-1 KD. PAR-3 KD cells also expressed more E-cadherin mRNA and protein. The enhanced adhesion kinetics of PAR-3 KDs was almost fully inhibited by calcium chelation, or by a HAV-motive decapeptide that affects E-cadherin intermolecular interactions. We propose that the enhanced rate of adhesion of PAR-3 KDs results from enhanced expression of E-cadherin, leading to a greater adhesion of free-floating cells to cells rapidly bound to the surface via their integrins, and particularly ITGαv.

## Introduction

Cell adhesion to basal membrane is one of the most important factors in targeted migration during development, as well as in cancer cells invasiveness and metastasis. Adhesion is of paramount importance to the three stages of cancer cells metastasis – detachment of the cell from the primary tumor, it's migration on the basement membrane, and the re-attachment of the migrating or blood-born cell to form a new secondary metastatic tumor. In the detachment and re-attachment stages, a fine balance has to be maintained between adhesion and migration in order to ensure the whole sequence of developmental or metastatic events [1], [2], [3].

Pancreatic adenocarcinoma (PAC) is one of the most aggressive human tumors, characterized by its propensity to rapidly metastasize [4], [5], [6]. PARs agonists, and particularly thrombin, have been implicated in invasion and metastasis [7], [8].

PANC-1 cell line is one of the more studied in vitro models of poorly differentiated human PAC. It has been very useful in studying PAC cells sensitivity to chemotherapeutic agents and has been, therefore, selected by our group for further detailed studies. We have recently reported that the knockdown of PAR-1 inhibits, while that of PAR-3 promotes PANC-1 cells invasiveness [9]. It was therefore of interest to examine the role of the two thrombin receptors, PAR-1 and -3, in PANC-1 cells adhesiveness. Since adhesion involves cell-surface interactions via integrins [10] and cell-cell interactions via cadherins [11], we studied the effects of PARs knockdown on the expression of these molecules.

We found that PAR-3 KDs exhibit faster adhesion kinetics than vector-control cells, whereas PAR-1 KDs did not exhibit any changes in adhesion. PAR-1 or PAR-3 KDs expressed higher levels of several integrins mRNAs, except for ITGαv, which exhibited increased mRNA and protein expression in PAR-3 KDs and decreased in PAR-1 KDs. PAR-3 KDs also expressed higher levels of E-cadherin. We propose that the higher expression of ITGαv and E-cadherin by PAR-3 KD cells is responsible for their altered adhesion properties.

## Materials and Methods

### Materials

#### PANC-1 cells were purchased from the ATTC (VA, USA)

Dulbecco's modified Eagle's medium (DMEM), fetal bovine serum (FBS), L-glutamine, penicillin/streptomycin, phosphate buffer saline (PBs), Hank's solution, and trypsin-EDTA solution were obtained from Biological Industries, Beit HaEmek, Israel. MTT was from Sigma (Petah Tiqva, Israel). Matrigel was from BD-Bioscience (Bedford, MA, USA). E-cadherin decapeptide inhibitor (FSHAVSSNG-NH2) was custom-synthesized by SBS Genetech, Beijing, China.

Anti-β-catenin (clone 14) was purchased from Cell Marque (Rocklin, CA, USA). For immunofluorescence, primary mouse monoclonal to CDH1 (E-cadherin, HECD-1) antibody was purchased from Abcam (Cambridge, MA, USA. DAPI and AlexaFluor secondary antibodies F(ab')2 fragment, 488 anti-mouse and 546 anti-rabbit were purchased from Invitrogen (Eugene, OR, USA). Integrin αV antibody was purchased from Cell-Signaling (Danvers, MA). GAPDH antibody was purchased from Abcam (Cambridge, UK). Secondary antibodies were purchased from LiCor (Lincoln, NE).

### Methods

#### Cell culture

cells were routinely cultured in DMEM, 10% fetal bovine serum (FBS), penicillin and streptomycin (50 U/ml and 50 μg/ml, respectively) at 37° and in 6/94% CO_2_/air mixture. Cells were re-fed twice each week

#### Knockdown cells

Stable PAR's knockdown cells were established in our laboratory essentially as previously described [12].

#### Adhesion assay

Cells were incubated in 96 wells plate, tissue culture-treated or coated with 0.2 mg/mL Matrigel, for the indicated times in medium containing 10% FBS. At the end of incubation, the medium containing non-adherent cells was transferred to a new well for overnight incubation. Adherent cells number was estimated by MTT assay (0.05 mg/ml). Reduced MTT absorbance was measured at 570 nm.

#### "Wound closure" assay

Cells were grown in 24 wells plate until ∼80% confluence. A cell-free “wound” was made by a scratch line and the initial size of the gap was measured. The kinetics of the of the wound closure was measured by examining timed micrographs of predetermined locations at 24, 48, 72 h. Closure kinetics was calculated as percent of the original width of the “wound” covered by newly attached cells.

#### PCR

Total RNA was extracted from two combined wells of 6 wells plate or from 25 cm flask using EZ-RNA-II kit (Biological Industries, Bet HaEmek, Israel) according to the manufacturers' protocols. RNA quality and purity was monitored by 260/280 nm OD ratios. High Capacity Reverse Transcription kit, Universal PCR Master Mix, and Taqman sequence-specific primers were from Applied Biosystems (Foster City, Ca, USA).

Real time-PCR was performed in 25 μl reaction volumes in 96-well plates using cDNA prepared from 1 μg of total RNA. Quantitative RT-PCR results were normalized to GAPDH.

#### Immunocytochemical staining

Cells were grown on Superfrost Plus glass slides (Menzel, Brunschweig, Germany). The slides were rinsed with Hank's solution and fixed for 3 h in 4% paraformaldehyde in Hank's solution.

Immunocytochemical stain was performed on the BenchMark XT (Ventana) using standard protocol (60 min pretreatment with CC1, blocking with I-View Inhibitor, 40 min/37°C 1st Ab and detection with SA-HRP/DAB).

#### Immunofluorescence staining

Cells (92,000/well) were grown overnight on Mat-Tek glass bottom (#1.5 poly-d-lysine coated) culture dishes (Mat-Tek Corp, Ashland, MA, USA). Cells were fixed overnight with 4% paraformaldehyde, washed with PBS, permeabilized with 0.1% SDS in PBS for 5 min and washed with PBS. Samples were blocked with donkey serum (5%) for 30 min, then incubated with primary antibodies for 1 h at 37°, rinsed extensively, exposed to secondary antibodies for 1 h at 37°, rinsed again and mounted with Mowiol plus DAPI, as a nuclear counterstain. Confocal micrographs were acquired on a Zeiss NLO META system using a 40× Plan-Apochromat 1.3NA objective. Detector gains remained constant for all acquisitions.

#### Western analysis

Cells were lysed in RIPA buffer (50 mM Tris, pH 8.0/150 mM NaCl/1.0% Nonidet P-40/0.5% Deoxycholate/0.1% SDS/0.2 mM NaVO_4_/10 mM NaF/0.4 mM EDTA/10% glycerol) with protease inhibitors (Roche, Mannheim, Germany). Lysates were sonicated for 20 s on ice and centrifuged at 10,000×*g* for 5 min. Lysates were then boiled for 5 min with Laemmli loading buffer followed by electrophoresis on 10% SDS polyacrylamide gels. Western analyses were performed with the recommended antibody dilutions. The blots were scanned using the LiCor laser-based image detection method.

#### Statistics

All experiments were performed several times in triplicates or quadruplicates. Student's t-test was used and differences were considered significant when p≤0.05.

## Results

### PAR-3 KDs adhere faster than vector-control cells

**Figure 1:**
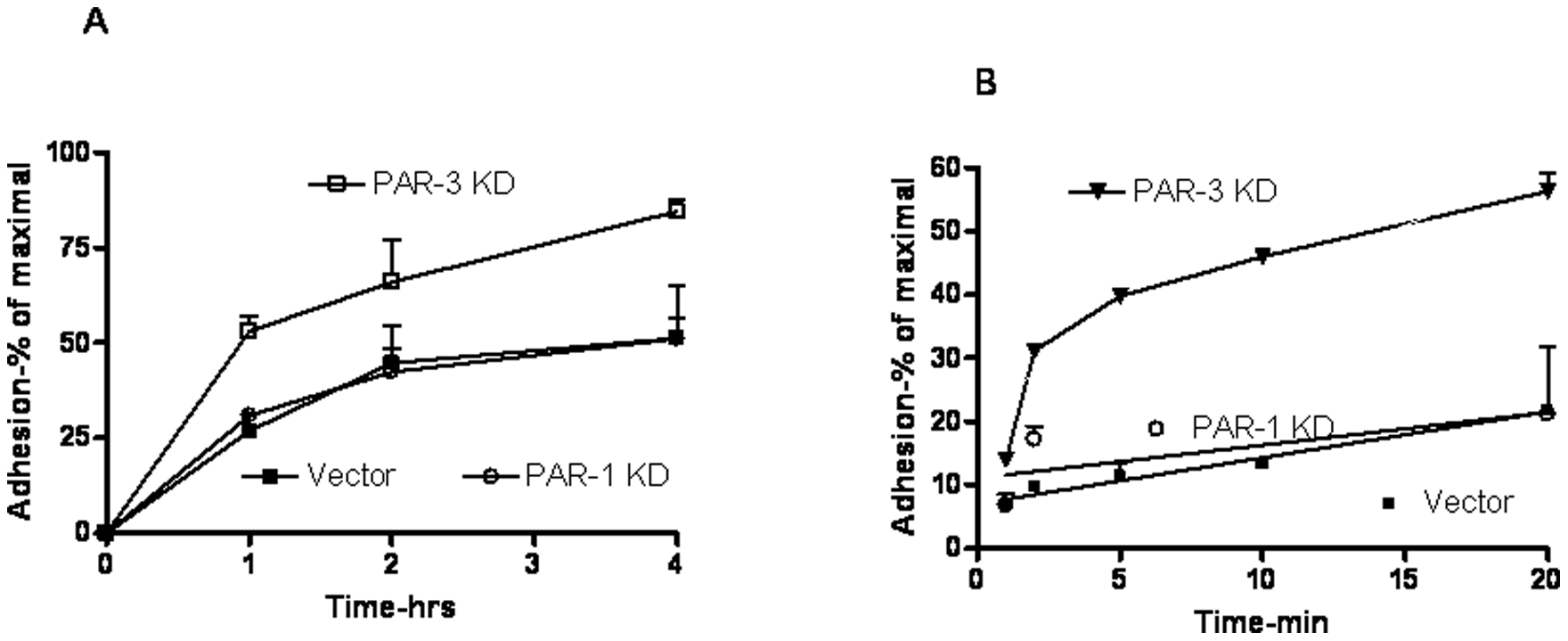
Adhesion kinetics of vector-control and PARs KDs cells. Cells of the desired PANC-1 variants were seeded at 10,000/well in a 96-wells cluster as described in Methods. At the indicated time points non-adherent cells were removed to a new well and the adherent cells number quantified by the MTT assay. All values were normalized to maximal adhesion (overninght incubation, usually >90% adhesion). A – adhesion kinetics to tissue-culture-treated clusters; B – Adhesion kinetics to Matrigel-coated (0.2 mg/ml for 1 h). Results represent mean±SE of 3–5 independent experiments performed in triplicates or tetraplicates.

We tested the rate of adhesion of vector-control, PAR-1 and PAR-3 KDs. Vector-control cells and PAR-1 KDs exhibited the same rate of adhesion to tissue culture-treated plasticware. Both variants exhibited rapid initial adhesion rate (approximately 30–35%/hr), and slower rate thereafter, reaching 50% adherent cells at 4 h of incubation (Fig. 1A). Both vector-control and PAR-1 KD cells exhibited more than 90% adhesion upon overnight incubation, which was defined as maximal. PAR-3 KDs adhered markedly more rapidly, reaching 60% adhesion at 1 h and more than 80% adhesion after 4 h (Fig. 1A).

**Figure 2:**
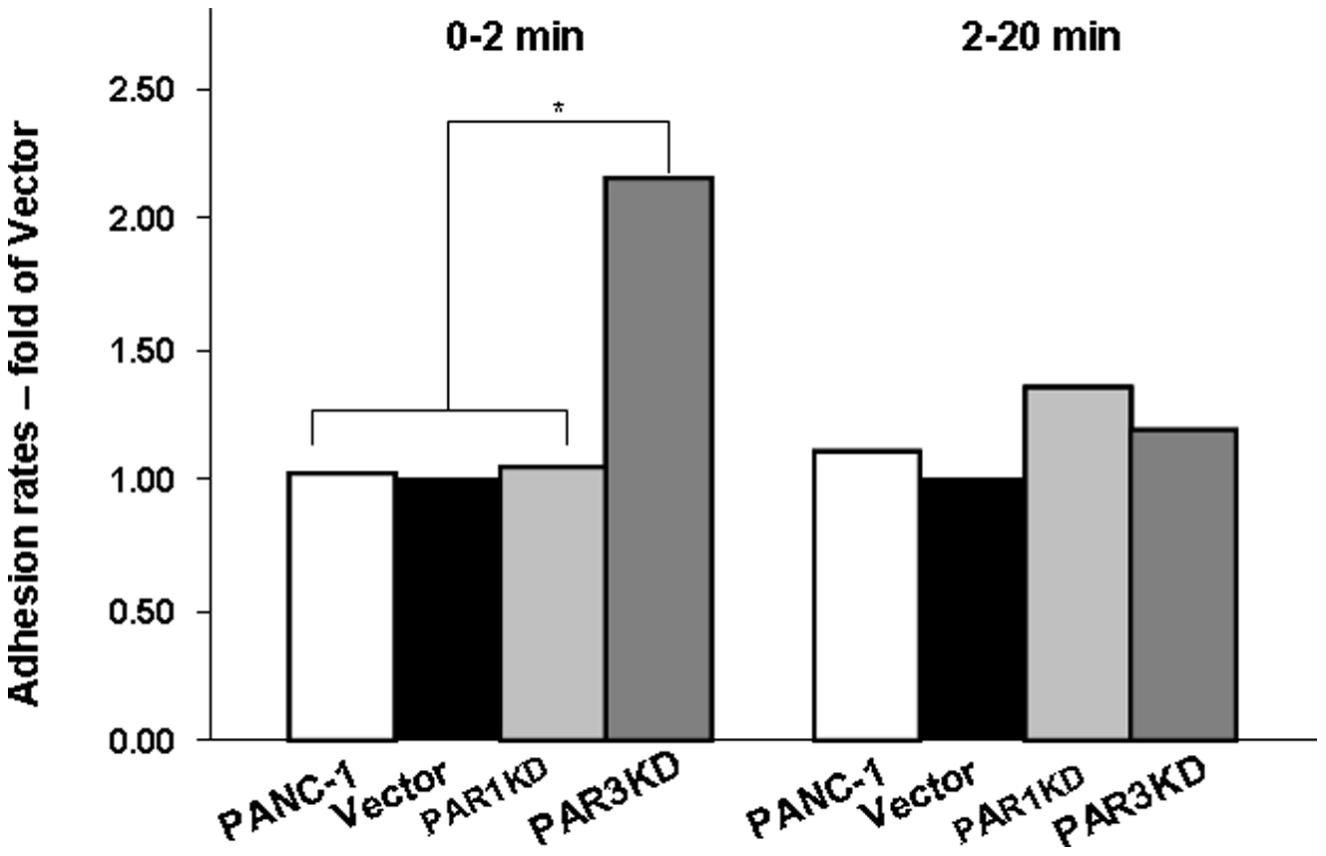
Increased adhesion of PAR-3 KD cells due to initial rate. Experiments were performed as described in Legend to Fig. 1B. The bars represent ratios of 0 to 2 min or 2 to 20 min adhesion rate values of the indicated PANC-1 variants to those of vector-control cells. The data are presented as ratios of averages obtained from four independent experiments. * denotes p<0.02, all other differences not significant.

**Figure 3:**
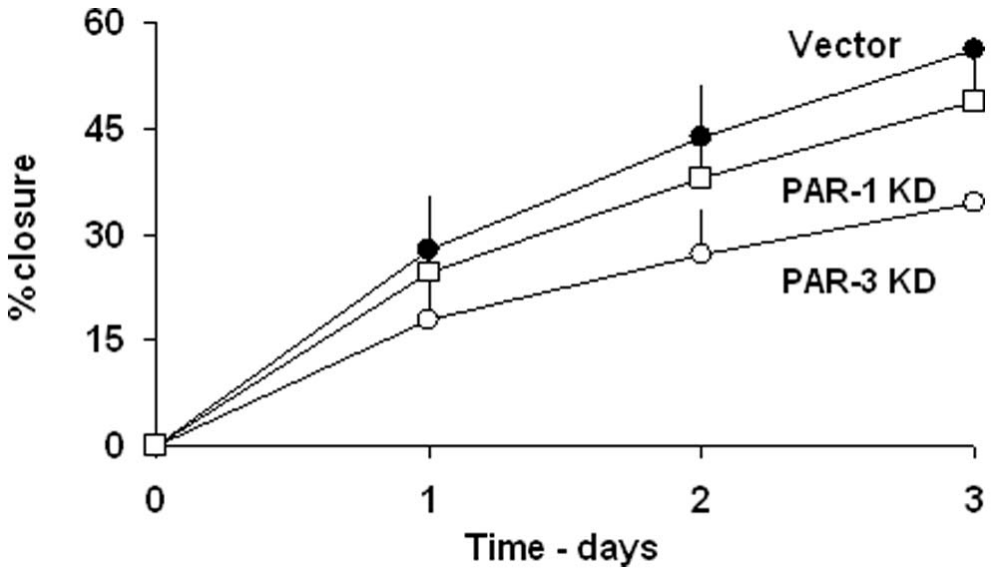
The effects of PARs knockdown on wound closure on wound closure kinetics. Vector control and the desired PARs knockdown cells were seeded at approximately 80% density. The kinetics of wound closure were performed as described in Methods. The results represent mean±SE of 5 independent experiments performed in triplicates. P<0.05 for Vector vs. PAR-3 KD and >0.05 vs. PAR-1 KD.

To examine the adhesion properties to a more physiological substrate, we tested the adhesion of PAR-1 or PAR-3 KDs and vector-control cells to plasticware coated with 200 μg/ml Matrigel. The kinetics of adhesion of vector-control cells was approximately twice faster than to non-coated plastic. PAR-1 KDs, PAR-3 KDs, however, adhered more rapidly, reaching 40% adhesion at 5 min incubation (Fig. 1B). The differences in adhesion rates could be clearly attributed to the initial, rapid adhesion (Fig. 2). The enhanced rate of adhesion of PAR-3 KDs was also reflected in a modest decrease of two-dimensional migration in an *in vitro* “wound closure” assay, while the rate of PAR-1 KDs was the same as that of vector-control cells (Fig. 3).

We interpreted these results as reflecting the non-homogeneity of the PANC-1 (and its variants) cell population, in which a sub-population of cells may exhibit different kinetics of adhesion due to putative differences in expression of adhesion molecules.

### PAR-1 and -3 KDs express higher integrins levels

To test this hypothesis, and to assess whether ITGs could be potentially involved in the very rapid adhesion rate of PAR-3 KDs, we assayed the expression levels of various integrins in PAR-3 KDs. Integrins (ITGs), the hetero-dimeric transmembrane adhesion proteins, play a major role in cells adhesion to extracellular matrix [10]. Since it is well documented that increased levels of integrins result in increased adhesiveness [13], we tested whether the enhanced rate of PAR-3 KDs adhesion reflects changes in the expression of an integrin or integrins. We examined the mRNA expression levels of several ITGs of the alpha and beta families in a sub-population of rapid adherents, i.e. cells adhering in 2 min (the time interval which showed the greatest differences between the kinetics of PAR-3 KDs and vector control cells) and compared them with those non-adherent after 20 min of incubation (slow adherents sub-population). The rapidly adhering cells were greatly enriched in mRNAs of ITGαv, ITGα6, and ITGα10 (not shown), suggesting that the rapid adhesion of this sub-population reflects the increased expression of one or more of these three ITGs.

**Figure 4:**
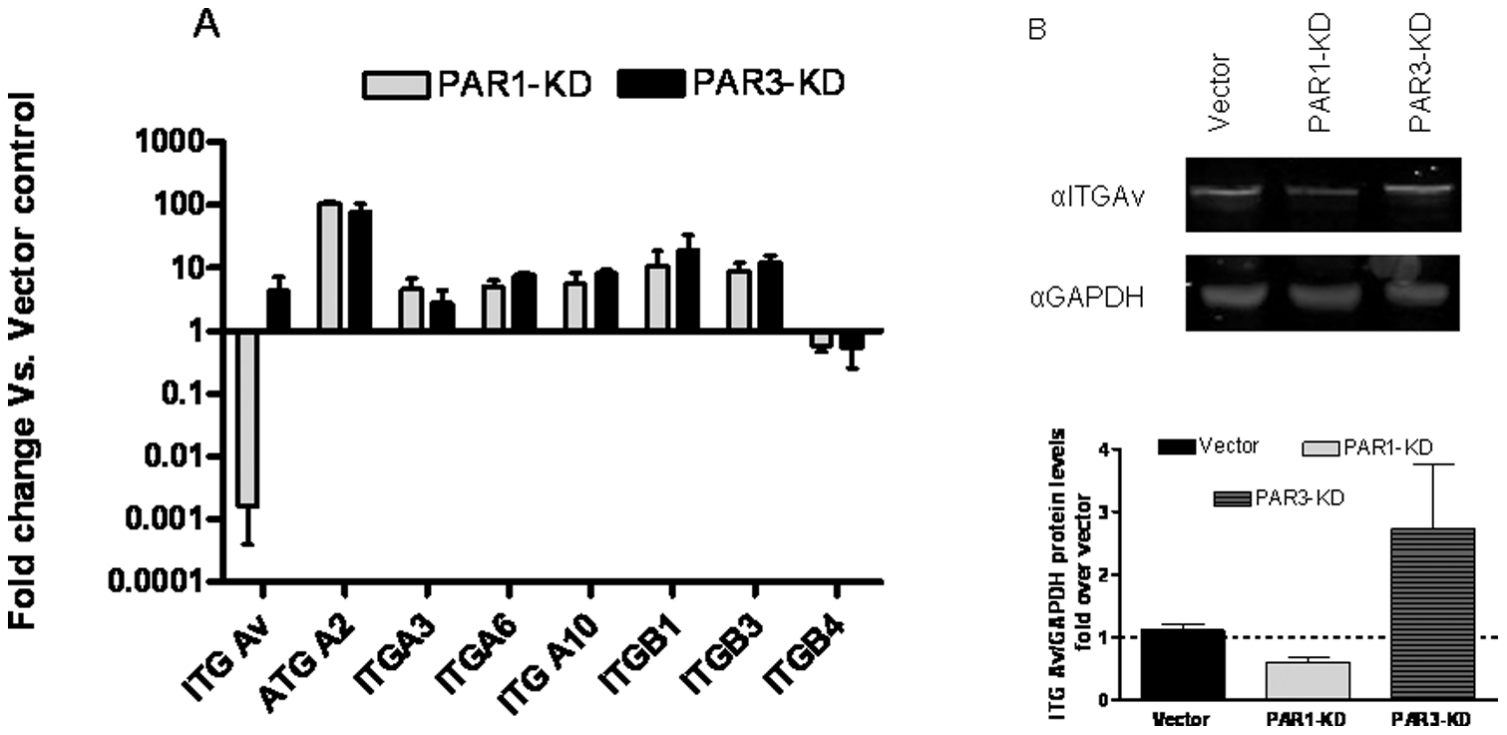
Integrins' mRNAs levels of vector-controls and PAR-1 or -3 KD cells. A- Vector-control, PAR-1 or -3 KD cells were assayed for mRNA levels of the designated integrins and normalized to GAPDH mRNA levels. The results are presented as fold difference of respective KDs mRNA levels to those of vector-controls. The results represent mean±SE of at least 3 independent experiments. B-Representative western blot analysis of ITGαV and GAPDH in Vector-Control, PAR-1 or -3 KD cells. Lowe panel-quantification of protein expression from three different western blots with 3 different cell lysates for each sample. The results represent mean±SE of 3 independent experiments.

To test whether the expression of ITGs could explain the adhesion differences between PAR-3 KDs and Vector controls or PAR-1 KDs, we examined their mRNAs expression levels in PAR-1 and -3 KDs and compared them to those in vector-control cells. Fig. 4A shows that both PAR-1 and PAR-3 KDs express higher levels of ITGα2, 3, 6, and 10, as well as ITGβ1 and 2 mRNAs. The increases ranged between 3- to over 100-fold of the levels assayed in vector-control cells. ITGβ4 mRNA exhibited a modestly decreased level in both KDs (Fig. 4A). These changes, common to both knockdowns, could not therefore account for the higher rate of adhesion of PAR-3 KDs. ITGαv mRNA levels, however, were affected differently in the two KDs. While in PAR-1 KDs ITGαv mRNA decreased by more than 99%, in PAR-3 KDs it increased almost ten-fold (Fig. 4A). Indeed, assaying ITGαv protein by Western analysis revealed a qualitatively similar picture, with a moderate decrease in PAR-1 KDs and an increase in PAR-3 KDs (Fig. 4B).

These results were compatible with the hypothesis that the increase in the expression of ITGαv might be responsible for the enhanced rate of adhesion of PAR-3 KDs.

### PAR-3 KD's express higher E-cadherin levels

**Figure 5:**
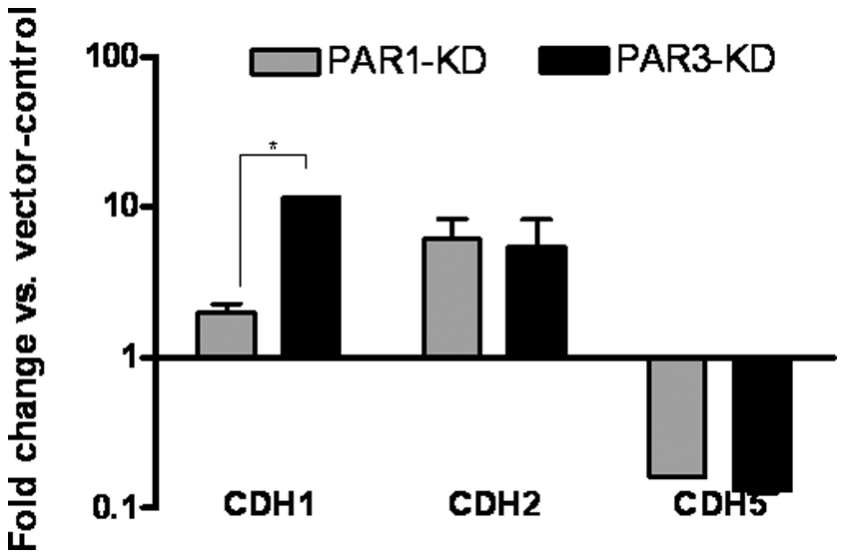
Cadherins' mRNAs levels of vector-controls and PAR-1 or -3 KD cells. Vector-control, PAR-1 or -3 KD cells were assayed for mRNA levels of the designated cadherins and normalized to GAPDH mRNA levels. The results are presented as fold difference of respective KDs mRNA levels to those of vector-controls. The results represent mean±SE, * denotes p<0.02

We next investigated the expression of cadherins. We postulated that the few rapidly adhering cells might serve as anchors for additional floating cells, “piggy-backing” via cadherin molecules. We found that PAR-1 and PAR-3 exhibited a 10-fold enrichment in CDH-2 and a decreased expression of CDH-5 mRNAs, whereas CDH-1 mRNA was more than 10-fold enriched in PAR-3 KDs and unchanged in PAR-1 KDs (Fig. 5).

**Figure 6:**
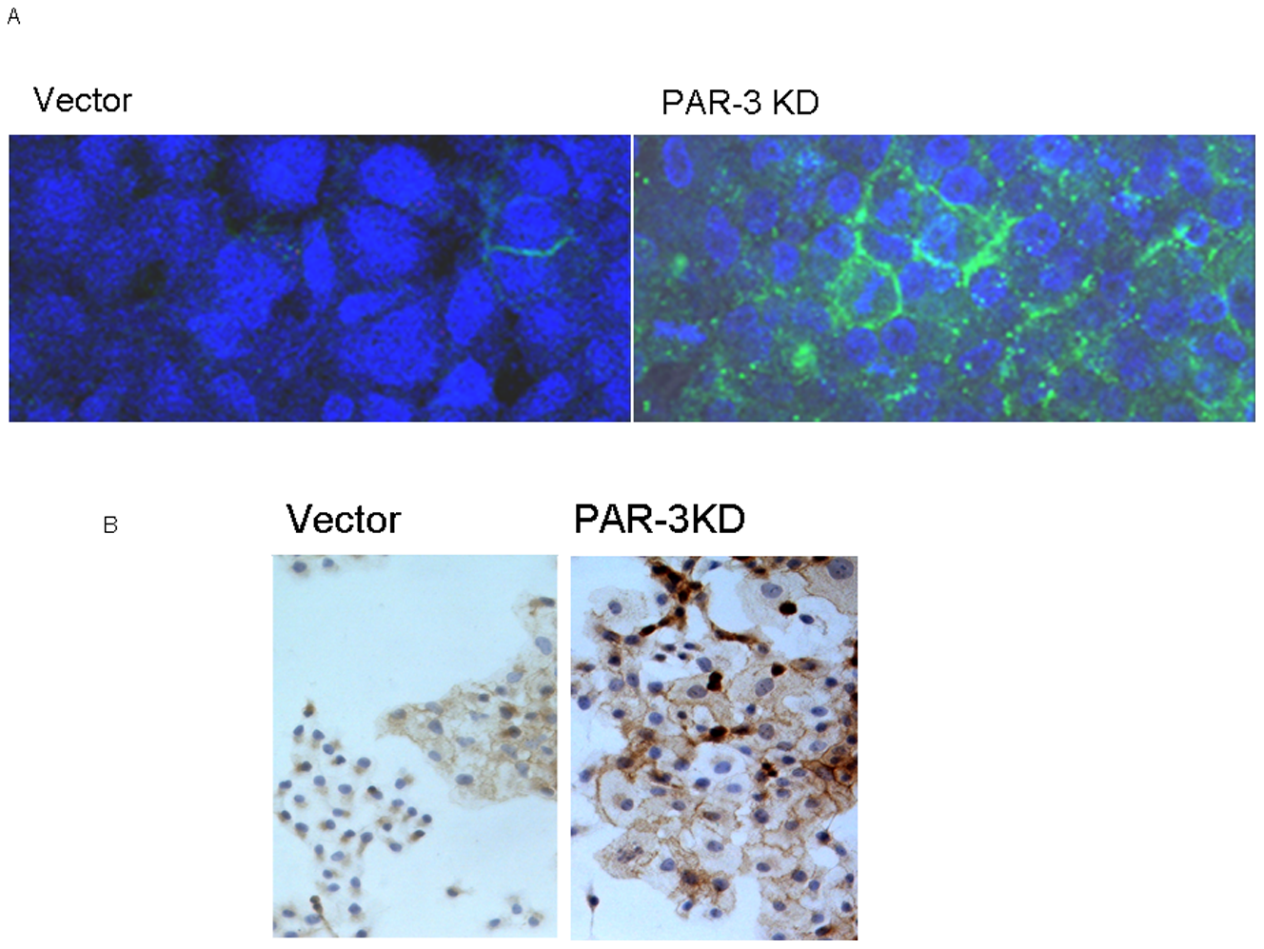
A- CHD1 and beta-catenin expression in Vector-control and PAR-3 KD cells. A- CHD1 immunofluorescent staining of Vector-control and PAR-3 KD cells Vector-control and PAR-3 KD cultures were stained for e-cadherin immunofluorescence and photographed using 25× objective as described in Methods. Blue- DAPI nuclear counterstain. **B-** Beta-catenin immunocytochemical staining. Vector-control or PAR-3 KD cultures were processed and stained for beta-catenin as described in Methods. Photomicrographs were acquired using ×4 objective.

To test whether the expression of CDH-1 protein was indeed increased in PAR-3 KDs, we performed immunocytochemical and immunofluorescence assays of this protein. Indeed, PAR-3 KDs exhibited a significant number of CDH-1 immunoflurescent cells (as opposed to practically none in vector-controls, Fig. 6A). In Fig. 6B we show that PAR-3 KDs cultures stained more for CDH-1 (as well as for β-catenin). Indeed, there were five-fold more cells strongly staining for E-cadherin in the PAR-3 KDs monolayer than in the vector-control monolayer (not shown).

**Figure 7:**
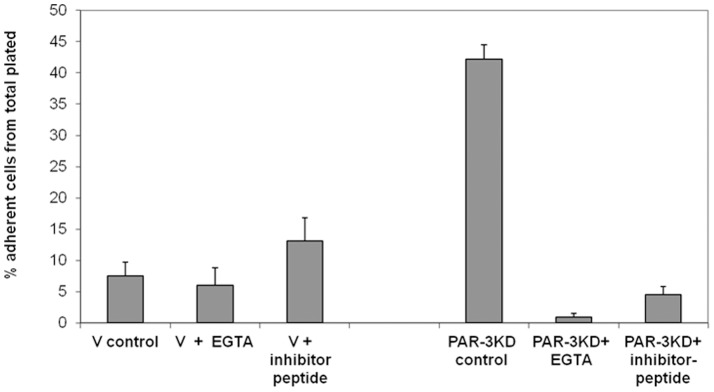
The effect of E-cadherin inhibition on adhesion. Vector-control or PAR-3 KD cells were plated in 96-wells clusters at 25,000 cells/well and allowed to adhere for 30 min. EGTA (5 mM) or HAV inhibitor peptide (1 mM) were added at time 0.

To test the hypothesis that the increased expression of E-cadherin was responsible for the more rapid kinetics of PAR-3 KDs adhesion, we included in the adhesion assay either 2.5 mM EGTA (to chelate calcium ions), or 1 mM of the HAV-motive decapeptide (previously shown to inhibit E-cadherin inter-molecular interactions, [14]). Both treatments greatly reduced the rapid adhesion of PAR-3 KD cells and had no effect on the adhesion of vector-controls (Fig. 7).

## Discussion

The adhesion properties of cancer cells determine their ability to detach from the *in situ* primary tumor, modulate the rate of their migration, and determine their re-attachment at the distant metastasis site. Except for the two extremes, i.e. no attachment or irreversible attachment, a change in adhesiveness may either increase or decrease the rates of detachment, migration, and/or re-attachment of cancer cells.

Pancreatic adenocarcinoma is one of the most aggressively metastatic tumors. There are a large number of reports suggesting that the expression or activation of PAR-1 contributes to the invasive charcteristics of cancer cells in general, and of pancreatic adenocarcinoma cells in particular [7], [15], [16], [17]. By comparison, the role(s) of the expression or activation of PAR-3 has not been studied in depth. Indeed, only a few reports demonstrated an independent signal transduction pathway for PAR-3 [18], while others suggested it serves as a PAR-1 co-receptor [19]. It was, therefore, interesting to investigate the effects of PAR-3 expression on the adhesion properties of pancreatic cancer cells and compare them to those of the better studied PAR-1, both thrombin-sensitive proteinase-activated receptors.

In our hands, knockdown of PAR-1 had little if any effect on the rate of adhesion of PANC-1 cells. PAR-3 KDs, however, exhibited a marked increase in the rate of adhesion to both plasticware and Matrigel coating. The knockdown of PAR-3 affected primarily the early kinetics of the adhesion process.

Indeed, the effects of PAR-1 or -3 knockdowns were compatible with their migration properties as reflected in the kinetics of *in vitro* wound closure assay. While PAR-1 KDs behaved like vector-infected controls, PAR-3 KDs exhibited markedly slower wound closure.

In order to elucidate the molecular mechanism(s) of this effect of PAR-3 knockdown, we investigated two families of molecules implicated in cell-matrix and cell-cell interactions: the integrins and the cadherins.

Comparison of PAR-1 and -3 KDs to vector-infected controls revealed an increased expression of all the tested integrins' mRNAs, with the exception of ITGαv mRNA, which exhibited a major (∼300-fold) decrease in PAR-1 KDs and an almost 10-fold increase in PAR-3 KD. Western analysis of vector-controls, and of PAR-1 or PAR-3 KDs confirmed these changes at the protein expression level. Moreover, when rapidly adhering PAR-3 KD cells were compared to the slowly adhering population, there was a 40-to-100-fold enrichment in ITGα10, ITGα6 and ITGαv mRNAs. These, results suggest that the increased rate of adhesion of PAR-3 KDs could be explained in terms of increased expression of ITGαv.

The lack of change in the rate of adhesion of PAR-1 KDs, despite the decrease in the level ITGαv mRNA, may reflect the increase in some or all other ITGs.

In order to explore the possibility that cell-cell adhesion contributes to the increase in cell-substrate adhesiveness of PAR-3 KD, we investigated the expression of cadherins in both PAR-1 and -3 KDs. While both knockdowns exhibited comparable increases in CDH2 and decreases in CDH5 mRNAs, CDH1 mRNA was markedly elevated in PAR-3 KDs and little change was found in PAR-1 KDs. We could thus predict a higher expression of E-cadherin protein in PAR-3 KDs. Indeed, immunohistochemistry and immunofluorescence staining confirmed this hypothesis.

Taking these results into account, we suggest that increased expression of ITGαv may promote rapid adhesion of a subpopulation of PAR-3 KD cells, which in turn cause secondary adhesion of a large population of cells via E-cadherin mediated interactions. The interplay between the integrins and the cadherins in cancer invasion and metastasis has been recently discussed by Canel *et al.* [*20*].

This hypothesis is strongly supported by our findings that calcium chelation or competition for E-cadherin-E-cadherin interactions by a HAV-motive decapeptide markedly inhibited adhesion of PAR-3 KDs, but had little if any effect on control cells.

Our data strongly suggest, but do not unequivocally prove, that the increased expression of ITGαv and CDH1 in PAR-3 KD is the primary change responsible for the enhanced adhesion kinetics of this PANC-1 variant. The cell-substrate and cell-cell adhesion processes are complex interactions in which multiple molecular species are involved, of which ITGαv and E-cadherin may be more important, but evidently not the sole species. Thus, vector-control cells present tight adhesive colonies despite very low expression of E-cadherin.

Our results suggest that the expression of PAR-3, a proteinase-activated receptor with few reported functions, is important in terms of expression of adhesion proteins and in the adhesion process itself. Our recent finding that knockdown of PAR-3 markedly enhances PANC-1 cells migration and invasion [9] is complementary to the findings presented here. Indeed, although the increased rate of adhesion of PAR-3 KDs modestly slows down two-dimensional migration in the “wound closure” assay, it may promote migration and adhesion in a three-dimensional system, reflecting a more physiological setting. Since these processes are vital to cancer metastasis, PAR-3 expression and function should be further investigated in additional *in vitro* and *in vivo* model systems.
